# Morphological and morphometric anatomy of the lesser occipital nerve and its possible clinical relevance

**DOI:** 10.1038/s41598-024-55648-8

**Published:** 2024-03-10

**Authors:** Latif Saglam, Osman Coskun, Ozcan Gayretli

**Affiliations:** Department of Anatomy, Istanbul Faculty of Medicine, Istanbul University, Millet Caddesi, Fatih, 34093 Istanbul, Turkey

**Keywords:** Lesser occipital nerve, Anatomy, Headache, Block, Decompression surgery, Anatomy, Neurology

## Abstract

The lesser occipital nerve (LON) has one of the most variations among occipital nerves. We aimed to investigate morphological and morphometric features of LON. A total of 24 cadavers, 14 males (58%) and 10 females (42%), were dissected bilaterally. LON was classified into 3 types. The number of branches and the perpendicular distances of the point where LON emerged from the posterior border of sternocleidomastoid muscle to vertical and transverse lines passing through external occipital protuberance were determined. The shortest distance between LON and great auricular nerve (GAN), and linear distance of LON to its branching point were measured. The most common variant was Type 1 (30 sides, 62.5%), followed by Type 2 (12 sides, 25%) and Type 3 (6 sides, 12.5%), respectively. In males, Type 1 (22 sides, 78.6%) was the most common, while Type 1 (8 sides, 40%) and Type 2 (8 sides, 40%) were equally common and the most common in females. On 48 sides, 2–9 branches of LON were observed. The perpendicular distance of said point to vertical and transverse lines was meanly 63.69 ± 11.28 mm and 78.83 ± 17.21 mm, respectively. The shortest distance between LON and GAN was meanly 16.62 ± 10.59 mm. The linear distance of LON to its branching point was meanly 31.24 ± 15.95 mm. The findings reported in this paper may help clinicians in estimating the location of the nerve and/or its branches for block or decompression surgery as well as preservation of LON during related procedures.

## Introduction

The lesser occipital nerve (LON), which is one of the cutaneous branches of the cervical plexus, originates chiefly from the ventral ramus of C2 and occasionally C3^1^. The LON curves around the spinal accessory nerve and passes anterior to it^1^. Afterward, it ascends along the posterior border of the sternocleidomastoid muscle (SCM). Close to the cranium, it pierces the deep fascia and supplies the skin of retroauricular region^1^. It communicates with the great auricular and greater occipital nerves and the auricular branch of the facial nerve^1^. The auricular branch of the LON, which may arise from the greater occipital nerve (GON), innervates some part of the medial aspect of the auricle (the upper third of the skin) and communicates with the posterior branch of the great auricular nerve (GAN)^1^.

The LON is one of the nerves with the most variation among the occipital nerves and therefore may be difficult to identify during interventional procedures^2^. Although it supplies a small area, the complex anatomy of the nerve may be important for the success of block and decompression surgery in migraine, occipital neuralgia, and cervicogenic headache^2–4^.

The LON has different types and can differ even on the right and left sides of the same individual^2,5^. During surgery, it is essential not to overlook the branches of the LON that can result from such different types. Because of these different morphologic features, exposing the LON up to its terminal branches is recommended during surgery^5^.

Preservation of the LON during the facelift procedure^6^, microvascular decompression surgery for hemifacial spasm^7^, and the retrosigmoid approach^8^ are important for the development of occipital sensory disturbance that may result from LON injury. In addition, the anatomy of the region should be well known so that structures such as the GAN and spinal accessory nerve, to which the LON is adjacent, are not damaged during dissection^2^.

There are many studies related with the anatomy of the LON^2–6,9–18^. Some of these studies^6,9–11,13,14^ have focused on the morphological features of the nerve, while others^2,3,5,15,16,18^ have studied on its morphometric features. However, very few studies^4,12^ have examined both morphological and morphometric features of it in detail. Although there are so many studies^2–6,9–18^ on the nerve, the fact that LON block and decompression surgery did not provide improvement in some of the patients may suggest that the surgical anatomy of the LON is still not fully elucidated. This study aims to investigate in detail the morphological and morphometric features of the LON, which may contribute to the easy identification of the LON in invasive procedures and its preservation during some surgical procedures.

## Methods

The study was performed on 24 cadavers (14 males, 58%; 10 females, 42%; 48 sides) (aged between 52 and 79 years, mean 69.08 years) at the Department of Anatomy, Istanbul Faculty of Medicine, Istanbul University. There was no visible deformity or pathology on the specimens. The Clinical Research Ethics Committee of the Istanbul Faculty of Medicine, Istanbul University approved this study (IRB (institutional review board) no: 2022/1505). The informed consent of all body donors was obtained from the body subjects/and or their legal guardians/next of kin. Two experienced anatomists (L.S. and O.G.) carried out the dissections of the specimens.

### Dissection technique and measurements

The posterior scalp of each specimen was shaved before dissection. Then, in the prone position, the first incision starting approximately 5 cm above the external occipital protuberance (EOP) and passing through the EOP was made and progressed to the spinous process of the prominent vertebra. From both ends of this incision, 10 cm long incisions extending transversely to the sides were made. Skin and subcutaneous tissues were dissected and trapezius (TM), splenius capitis (SC), and sternocleidomastoid muscles were exposed. The point where the LON emerged from the posterior margin of the SCM was found and the course of the nerve was followed proximally and distally. Dissection was carried out very carefully to avoid damaging the nerve and its branches. Each LON was evaluated morphologically and the following classification was based on the obtained data.

### Classification

**Figure 1:**
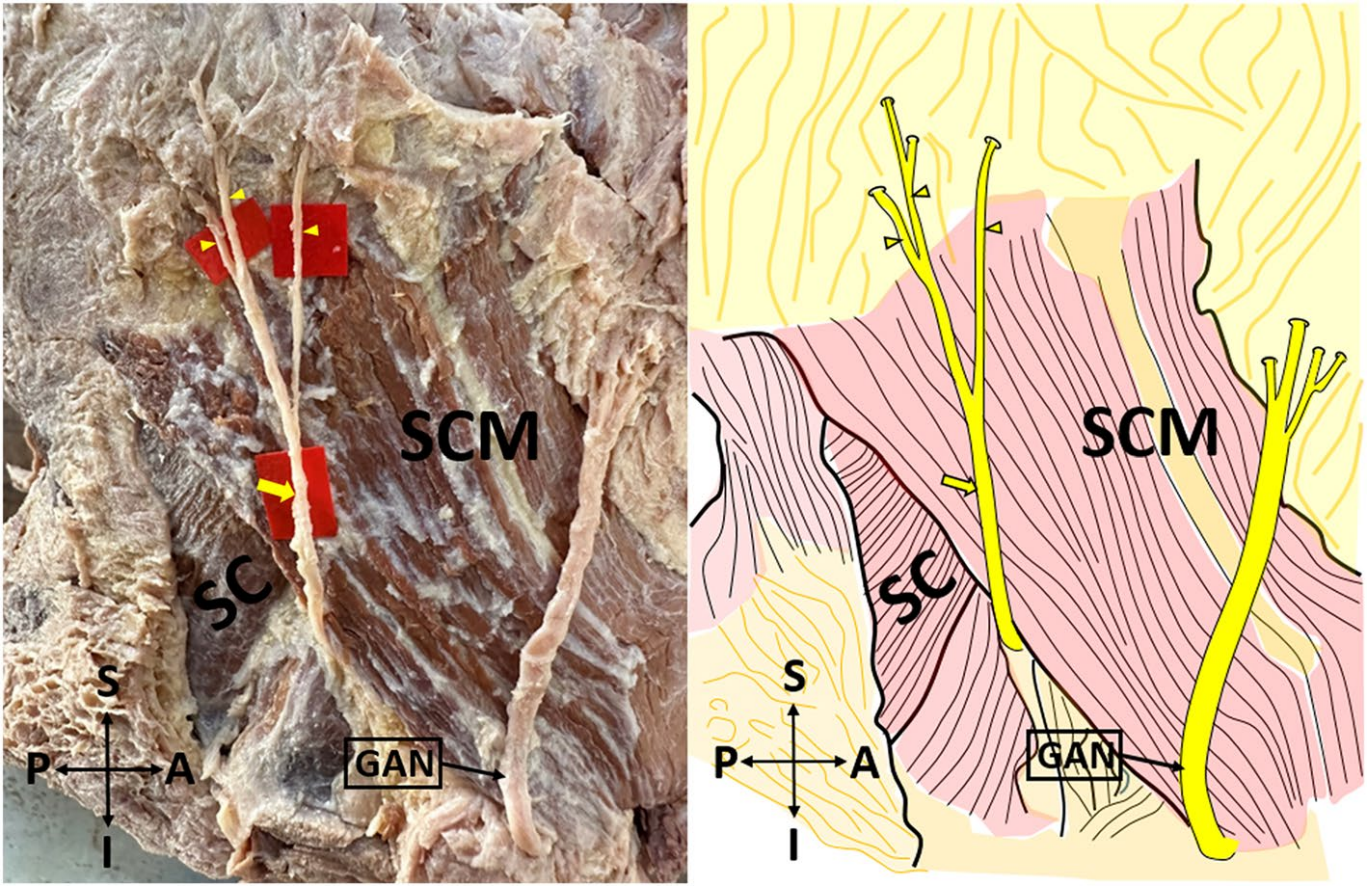
Type 1 lesser occipital nerve on the cadaver (on the left side), and its schematic demonstration (on the right side) (lateral view). *SCM* sternocleidomastoid muscle; *SC* splenius capitis muscle; *GAN* great auricular nerve; *A* anterior; *P* posterior; *S* superior; *I* inferior. The yellow arrow shows the lesser occipital nerve. Type 1: Lesser occipital nerve emerges from the posterior border of the sternocleidomastoid muscle, courses over this muscle, and divides into its terminal branches, some of these branches pass anteriorly over the sternocleidomastoid muscle.

*Type 1* emerges from the posterior border of the SCM, courses over this muscle, and divides into its terminal branches, some of these branches pass anteriorly over the SCM (Fig. 1).

**Figure 2:**
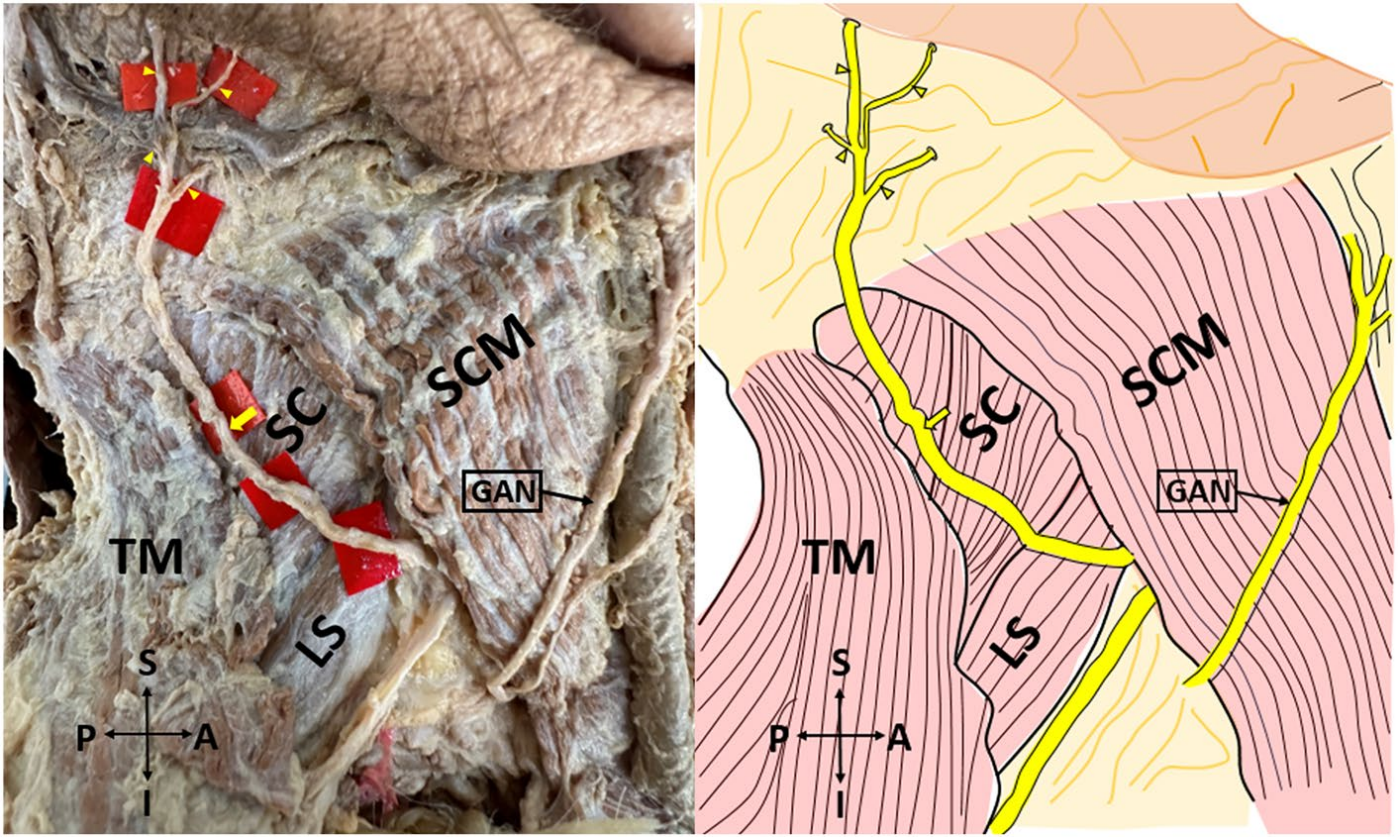
Type 2 lesser occipital nerve on the cadaver (on the left side), and its schematic demonstration (on the right side) (lateral view). *SCM* sternocleidomastoid muscle; *TM* trapezius muscle; *SC* splenius capitis muscle; *LS* levator scapulae muscle; *GAN* great auricular nerve, *A* anterior; *P* posterior; *S* superior; *I* inferior. The yellow arrow shows the lesser occipital nerve. The yellow arrowheads show the branches of the lesser occipital nerve. Type 2: Lesser occipital nerve arises from the posterior border of the sternocleidomastoid muscle, courses over splenius capitis and levator scapulae muscles, and branches into the scalp by passing between trapezius and sternocleidomastoid muscles.

*Type 2* LON arises from the posterior border of the SCM, courses over SC and levator scapulae muscle (LS), and branches into the scalp by passing between TM and SCM (Fig. 2).

**Figure 3:**
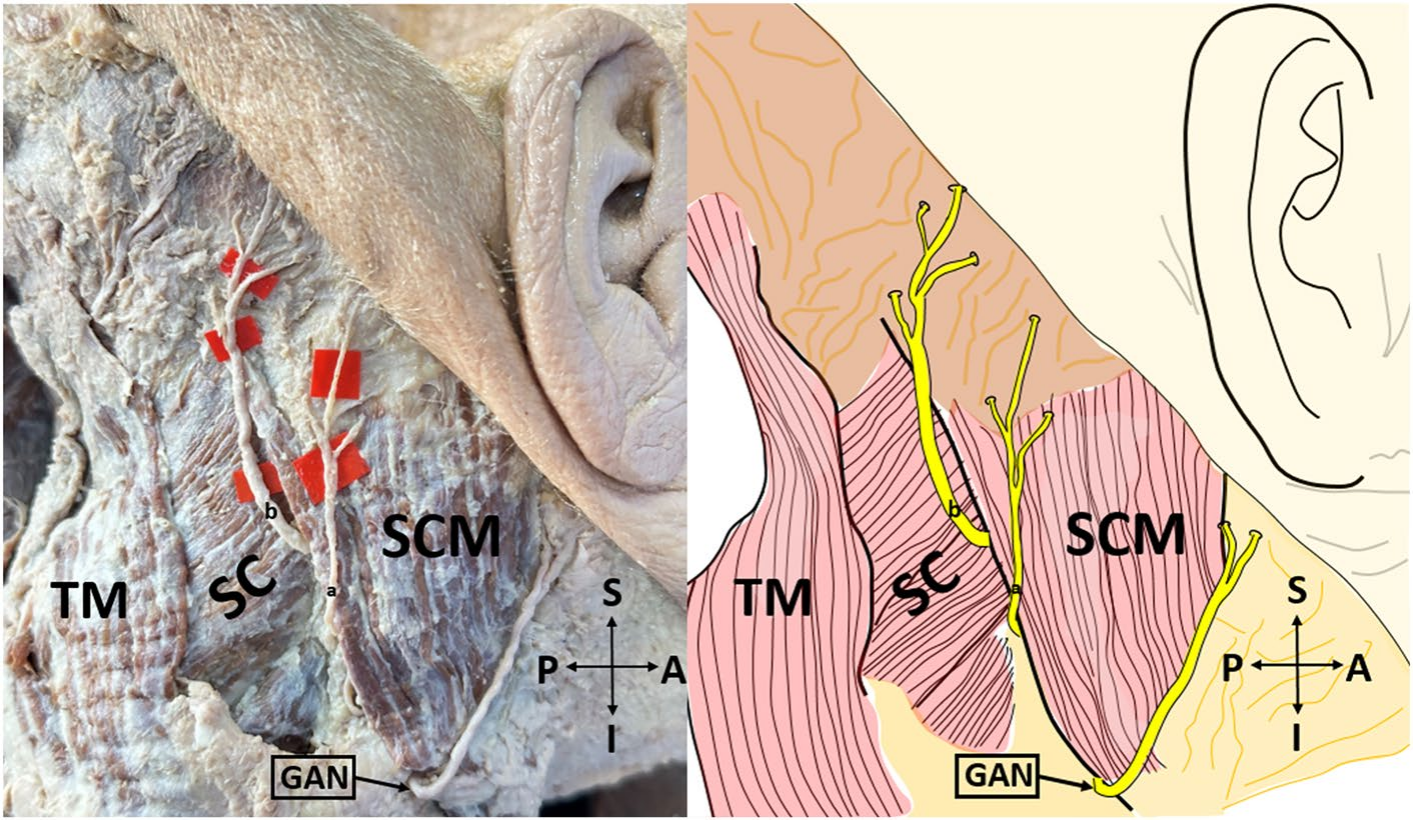
Type 3 lesser occipital nerve on the cadaver (on the left side), and its schematic demonstration (on the right side) (lateral view). *SCM* sternocleidomastoid muscle; *TM* trapezius muscle; *SC* splenius capitis muscle; *GAN* great auricular nerve, *A* anterior; *P* posterior; *S* superior; *I* inferior; a, one of the two main branches of the lesser occipital nerve; b, the other main branch of the lesser occipital nerve. Type 3: Lesser occipital nerve is split into 2 branches before emerging from the posterior border of the sternocleidomastoid muscle. One of these branches circles the posterior margin of the sternocleidomastoid muscle and courses over this muscle (**a**). The other branch circles the posterior margin of the sternocleidomastoid muscle and travels near the posterior border of the sternocleidomastoid muscle and then it runs on the sternocleidomastoid muscle and branches close to the sternocleidomastoid muscle's insertion (**b**).

*Type 3* LON is split into 2 branches before emerging from the posterior border of the SCM. One of these branches circles the posterior margin of the SCM and courses over this muscle. The other branch circles the posterior margin of the SCM and travels near the posterior border of the SCM and then it runs on the SCM and branches close to the SCM's insertion (Fig. 3). In addition, the number of branches of the LON was determined.

**Figure 4:**
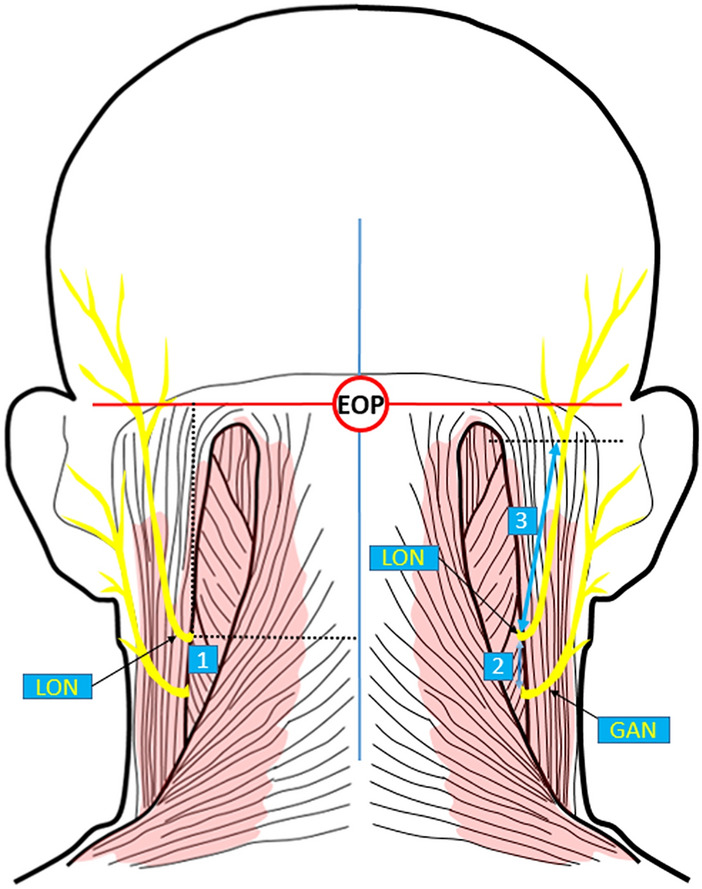
Demonstration of the morphometric measurements regarding the lesser occipital nerve (posterior view). *EOP* external occipital protuberance; *LON* lesser occipital nerve; *GAN* great auricular nerve, *A* anterior; *P* posterior; *S* superior; *I* inferior; 1, the point where the LON emerged from the posterior border of the SCM; 2, the shortest distance between the LON and the great auricular nerve; 3, the linear distance of the lesser occipital nerve from its emergence at the SCM to its branching point. Black dotted lines indicate the perpendicular distance of the 1 to the vertical and transverse lines passing through the EOP.

The vertical and transverse lines passing through the EOP were marked and the perpendicular distances of the point where the LON emerged from the posterior border of the SCM to these lines were calculated. Moreover, the shortest distance between LON and GAN, and the linear distance of the LON from its emergence at the SCM to its branching point was measured. All of the morphometric measurements are represented in Fig. 4.

A digital caliper accurate to 0.01 mm (INSIZE Co., Ltd., Taiwan) was used to measure the distances. All parameters were measured by the same researcher and repeated randomly 15 days later. If there was more than a 10% difference between the two measurements, the relevant measurement was repeated.

### Statistical evaluation

The data were assessed using IBM SPSS (The Statistical Package for the Social Sciences) version 21.0 package program. For descriptive analyses, categorical variables were defined as frequency (n) and percentage (%), and continuous variables were expressed as mean, standard deviation, or median (minimum–maximum). Normal distribution of continuous variables was performed using the Kolmogorov–Smirnov test. In the comparison of continuous variables of two independent groups; the Independent groups t-test was used if normally distributed, Mann–Whitney U test was used if not normally distributed. Univariate generalized linear model was utilized to evaluate the relationship of normally distributed continuous variables with more than two factors. Statistical significance was determined as *p* < 0.05.

### Ethics approval

This study was performed in line with the principles of the Declaration of Helsinki. Approval was granted by the Clinical Research Ethical Committee of Istanbul Faculty of Medicine, Istanbul University (Date: 09/09/2022, No: 16).

## Results

**Figure 5:**
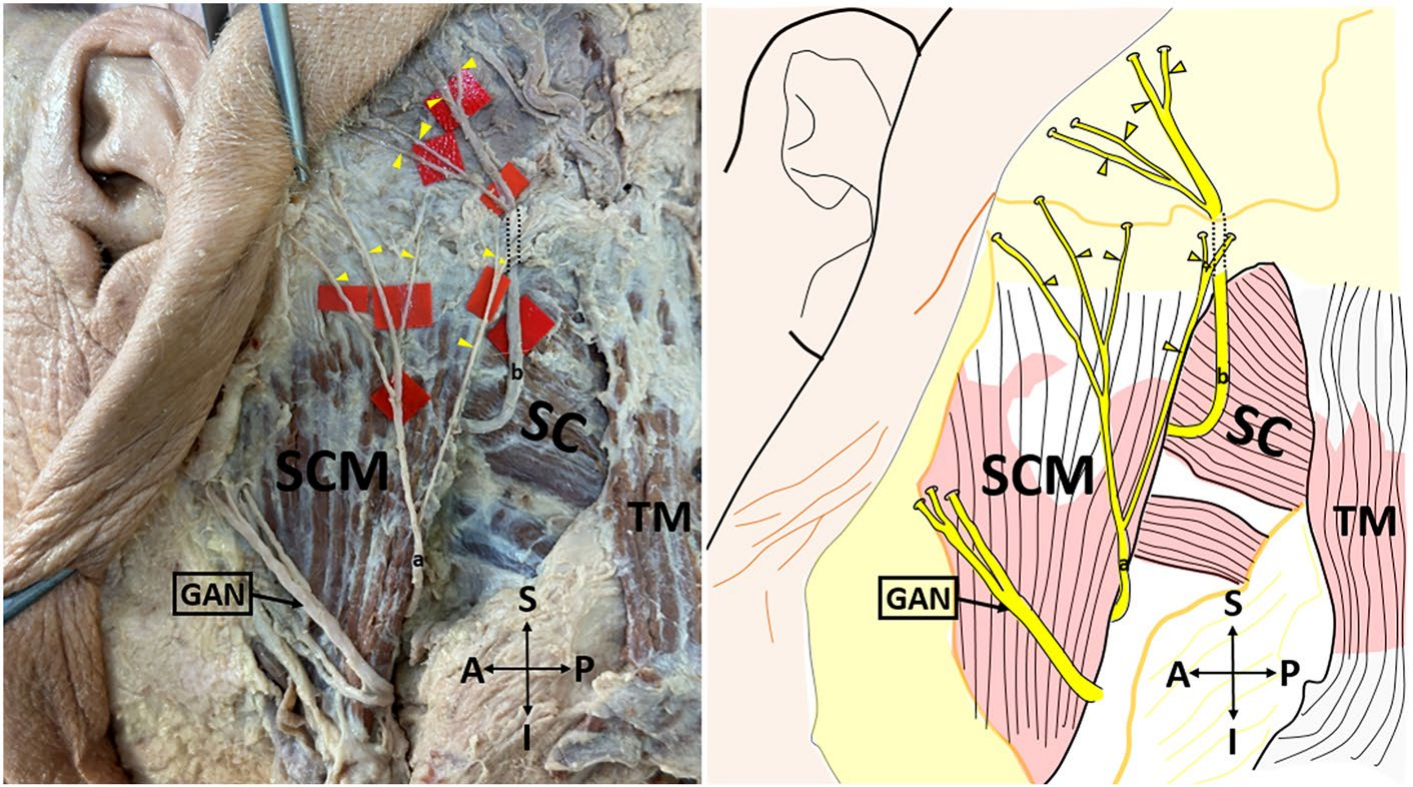
One of the main branches of the lesser occipital nerve passing through an aponeurotic tunnel adjacent to the superior part of the sternocleidomastoid muscle (on the left side), and its schematic representation (on the right side) (lateral view). *SCM* sternocleidomastoid muscle; *TM* trapezius muscle; *SC* splenius capitis muscle; *GAN* great auricular nerve, *A* anterior; *P* posterior; *S* superior; *I* inferior; a, one of the two main branches of the lesser occipital nerve; *b* the other main branch of the lesser occipital nerve that passes through an aponeurotic tunnel adjacent to the superior part of the SCM. The yellow arrowheads show the branches of the lesser occipital nerve. Black dotted lines show the boundaries of the said tunnel and the tunnel is crossed by one of the terminal branches of the other main branch of the lesser occipital nerve.

The rates of obtaining the types were Type 1 was on 30 sides (62.5%), Type 2 was on 12 sides (25%), and Type 3 was on 6 sides (12.5%), respectively. In males, Type 1 was the most common, while Type 1 and Type 2 were equally common and the most common in females. For the sides, Type 1 was the most common type for both sides. The detailed distribution of numbers and frequency of types of the lesser occipital nerve in terms of gender and side are shown in Table 1. On the left side of a specimen, one of the main branches of the LON was observed to pass through an aponeurotic tunnel adjacent to the upper part of the SCM. The tunnel was also crossed by one of the terminal branches of the other main branch of the LON (Fig. 5). Additionally, on a total of 48 sides, 2–9 branches of the LON were observed (median = 3).

**Table 1:** Distribution of numbers and frequency of types of the lesser occipital nerve in terms of gender and side.

	n (side)	Type 1	Type 2	Type 3
Male	28	22 (78.6%)	4 (14.3%)	2 (7.1)
Female	20	8 (40%)	8 (40%)	4 (20%)
Right	24	16 (66.7%)	5 (20.8%)	3 (12.5%)
Left	24	14 (58.3%)	7 (29.2%)	3 (12.5%)
Total	48	30 (62.5%)	12 (25%)	6 (12.5%)

*Type 1* Lesser occipital nerve emerges from the posterior border of the sternocleidomastoid muscle, courses over this muscle, and divides into its terminal branches, some of these branches pass anteriorly over the sternocleidomastoid muscle.

*Type 2* Lesser occipital nerve arises from the posterior border of the sternocleidomastoid muscle, courses over splenius capitis and levator scapulae muscles, and branches into the scalp by passing between trapezius and sternocleidomastoid muscles.

*Type 3* Lesser occipital nerve is split into 2 branches before emerging from the posterior border of the sternocleidomastoid muscle. One of these branches circles the posterior margin of the sternocleidomastoid muscle and courses over this muscle. The other branch circles the posterior margin of the sternocleidomastoid muscle and travels near the posterior border of the sternocleidomastoid muscle and then it runs on the sternocleidomastoid muscle and branches close to the sternocleidomastoid muscle's insertion.

The overall mean and standard deviation results of the measured values are shown in Table 2. Similarly, the results of the analysis in terms of side and gender are given in Tables 3 and 4, respectively. As a result of the Generalized Linear Model analysis, a statistical significance was observed between genders for the perpendicular distance of the LON to the vertical line passing from the EOP (*p* = 0.027).

**Table 2:** The overall morphometric data regarding the lesser occipital nerve (n = 48).

Parameters	Mean ± standard deviation (mm)
The perpendicular distance of the point where the LON emerges from the posterior border of the SCM to the vertical line passing from the EOP	63.69 ± 11.28
The perpendicular distance of the point where the LON emerges from the posterior border of the SCM to the transverse line passing from the EOP	78.83 ± 17.21
The shortest distance between LON and GAN	16.62 ± 10.59
The linear distance of the LON from its emergence at the SCM to its branching point	31.24 ± 15.95

*EOP* external occipital protuberance, *LON* lesser occipital nerve, *GAN* great auricular nerve, *SCM* sternocleidomastoid muscle.

**Table 3:** Analysis of morphometric data of the lesser occipital nerve in terms of side.

Parameters	Right (n = 24 sides)	Left (n = 24 sides)	*p* value
	Mean ± standard deviation (mm)	Mean ± standard deviation (mm)	
The perpendicular distance of the point where the LON emerges from the posterior border of the SCM to the vertical line passing from the EOP	64.92 ± 10.20	62.47 ± 12.37	0.459*
The perpendicular distance of the point where the LON emerges from the posterior border of the SCM to the transverse line passing from the EOP	79.08 ± 17.72	78.58 ± 17.06	0.922*
The shortest distance between LON and GAN	17.03 ± 10.76	16.20 ± 10.64	0.796*
The linear distance of the LON from its emergence at the SCM to its branching point	30.40 ± 15.37	32.07 ± 16.79	0.721*

*Independent samples t-tests were applied.

*EOP* external occipital protuberance, *LON* lesser occipital nerve, *GAN* great auricular nerve, *SCM* sternocleidomastoid muscle.

**Table 4:** Analysis of morphometric data of the lesser occipital nerve in terms of gender.

Parameters	Male (n = 28 sides)	Female (n = 20 sides)	*p* value
	Mean ± standard deviation (mm)	Mean ± standard deviation (mm)	
The perpendicular distance of the point where the LON emerges from the posterior border of the SCM to the vertical line passing from the EOP	66.75 ± 12.39	59.41 ± 7.98	0.025*
The perpendicular distance of the point where the LON emerges from the posterior border of the SCM to the transverse line passing from the EOP	78.35 ± 18.51	79.51 ± 15.64	0.821*
The shortest distance between LON and GAN	15.15 ± 10.92	18.89 ± 9.91	0.247*
The linear distance of the LON from its emergence at the SCM to its branching point	28.33 ± 16.12	35.31 ± 15.16	0.137*

*Independent samples t test were applied.

*EOP* external occipital protuberance, *LON* lesser occipital nerve, *GAN* great auricular nerve, *SCM* sternocleidomastoid muscle.

## Discussion

### Morphological features

The first morphological classification of LON appears in the study of De Araújo Lucas et al.^9^. However, they made a classification without using the concept of ‘type’. De Araújo Lucas et al. dissected a total of 8 cadavers (16 sides)^9^. They stated that, on the 9 sides, the LON was divided into two branches under the SCM. Afterward, one of these branches circled the posterior margin of the SCM, crossed this muscle, and ran towards the retroauricular region by passing over the posterolateral side of the mastoid process (MP). The other branch, similar to the first branch, circled the posterior margin of the SCM and traveled medial to the head by traversing the SCM from its most dorsal point. They also found that, on the 7 sides, the LON coursed over the LS and SC, and then passed between the TM and SCM. Pantaloni & Sullivan studied the LON on 19 hemifaces^6^. They observed that, on the 16 hemifaces, the LON arose behind the posterior edge of the SCM, it then progressed to MP and ear in an oblique-cephalic direction by running on the SCM fascia. On the remaining 3 hemifaces, the LON coursed over the SC and LS fascias. Madhavi & Holla^10^ presented a case report having the triplication of the LON and defined it as LON I-II-III. LON-I hooked around the posterior margin of the SCM and ran cranially, superficial to SCM. LON-II, a slender branch, emerged from the supraclavicular nerve trunk branch to the TM and coursed along the posterior border of the SCM parallel to LON-I. LON-III, which was thicker than other branches, arose from the posterior border of SCM, and coursed superomedially deep to the TM. Afterward, it perforated the fascia 3 cm inferior to the superior nuchal line and 3.5 cm lateral to the midline, and divided into two branches below this line. Ducic et al. investigated the LON on 112 live patients and 13 cadavers^4^. The vast majority of the LONs (85% of individuals) were settled along the posterior margin of the SCM, at a certain point 3 cm below the EOP. A smaller proportion of LONs (15% of individuals) were observed at different locations from the mid-superficial surface of the SCM muscle to within 1 cm of the GON. Sirasanagandla et al. stated a case report in which the LON's course was similar to the LON-III but pierced the TM fascia at a lower level^11^. Tubbs et al. dissected 10 cadavers and used the notion of ‘type’ for the first time^12^. They observed two types of LON. Type I which was observed in 60 percent of all specimens, referred to LONs that were coursed close to the posterior border of the SCM, and some of these passed anteriorly over the SCM near the MP. Type II, which was found to be less common than Type I (40% of total cadavers), was depicted as LONs that left from the posterior margin of the SCM and proceeded medially towards the occiput. In their subsequent study, Tubbs et al. dissected fifty-six cadavers (112 sides) to investigate the accessory nerve and observed that the LONs emerged directly from the accessory nerve on 2 sides (1.8%)^13^. Moreover, the LON communicated with the accessory nerve on 5.4% of the sides. Chabot et al. similarly reported a case in which the LON originated directly from the spinal accessory nerve^14^. In our study, the LON was classified into three types according to the observed morphological pattern. Type 1 (30 sides, 62.5%) which was the most common type in this study, referred to the LON that emerged from the posterior border of the SCM, coursed over this muscle, and divided into its terminal branches, some of these branches passed anteriorly over the SCM (Fig. 1). Type 2 (12 sides, 25%) was the LON, that arose from the posterior border of the SCM, coursed over SC and LS, and branched into the scalp by passing between TM and SCM (Fig. 2). Type 3 (6 sides, 12.5%), which was the least common seen in our study, the LON was split into 2 branches before emerging from the posterior border of the SCM. One of these branches circled the posterior margin of the SCM and coursed over this muscle. The other branch circled the posterior margin of the SCM and traveled near the posterior border of the SCM and then it ran on the SCM and branched close to the SCM's insertion (Fig. 3). Our Type 1 results are compatible with the results of Tubbs et al.^12^, while Type 2 and Type 3 results are inconsistent with the results of previous studies. Different sample sizes and/or races may explain this discrepancy. Peled et al. emphasized that the branching pattern of the LON was quite variable so it was important not to miss any branches during surgery^5^. Consequently, therefore, they emphasized that the LON should be dissected up to its distal part during the applications. Moreover, they reported that the morphology of LON was inconsistent, and may even vary from one side of the neck to the other^2,5^. Type 1 and Type 2 groups were obtained at the highest rates, respectively, in our study. According to our results, the rate of encountering Type 1 and Type 2 groups during LON-related surgery is quite high. In this context, we think that dissection and identification of these types during surgery is easier than Type 3. In particular, it can be said that block and decompression applications for LON may be easier in these groups. That being said, although the Type 3 group was the least common, it still represented a significant proportion (12.5%). In the Type 3 group, we believe that it is important to know that there are 2 emergence points behind the SCM in terms of the success of the block and decompression applications planned to be performed at this point.

Branches of the LON may be squeezed by fascial bands during their course, at the nuchal line, or at points where they interact with branches of the occipital artery^5,15^. In our study, it was found that on the left side, one of the main branches of the LON passed through an aponeurosial tunnel in the upper part of the SCM (Fig. 5). Although it was observed on only one side, we think it may be important for the success rate of invasive procedures for the LON. In terms of identifying these structures, we believe that further studies with a larger sample size should be conducted. Pantaloni & Sullivan examined the LON on the 19 hemifaces and observed that the 13 were divided into two branches, 3 into three, 2 into four, and 1 into five branches^6^. They also found that the two LONs were double. Khavanin et al. noted that this number of branches was four or more^2^. In the current study, the number of branches of the LON was a median of 3 (range: 2–9 branches). Each branch of the LON may be a potential compression point, so these branches should not be missed during surgical procedures^5^. We think that the knowledge that LON can branch up to 9 branches may be useful in surgical applications for the LON. Thus, complications caused by missing any branch of the nerve may be prevented.

### Morphometric features

In morphometric studies regarding the LON, researchers^2,3,5,15,16,18^ have utilized different certain landmarks for the measurements. Consequently, no consensus has been provided for measurements of the LON. Dash et al. dissected 20 cadaver heads and found a total of 30 LONs^3^. They calculated the perpendicular distances of the point where the LON emerged from the posterior margin of the SCM to the posterior midline and the transverse line passing through the lowest points of the external auditory canals were meanly 65.4 ± 11.6 mm and 53.3 ± 15.6 mm, respectively. They also observed that 4 LONs (13.3%) pierced the SCM. Tubbs et al. studied 12 adult formalin-fixed cadavers and observed that the main LON trunk was meanly 7 cm lateral to the EOP; similarly, the LON was meanly 3 cm medial to the tip of MP^16^. They also found that the LON split into its medial and lateral components at approximately the midpoint of the perpendicular distance between the transverse line through the EOP and the intermastoid line. Ducic et al. examined 112 live patients and 13 cadavers and found that the LON was settled along the posterior margin of the SCM, at a reference point 3 cm below the EOP in 85 percent of individuals^4^. They also reported that in 15 percent of individuals, the location of the nerve ranged from the relative midpoint of the SCM to within 1 cm of the GON. Lee et al. used a total of 10 fresh cadavers to investigate LON^15^. They found the perpendicular distances of the point where the LON arose from the posterior border of the SCM to the posterior midline (y axis) and the transverse line passing through the most anterosuperior points of the external auditory canals (x axis) were meanly 6.4 ± 1.4 cm and 0.5 ± 0.9 cm, respectively. Peled et al. dissected eight fresh frozen cadavers and found a total of 15 LONs^5^. They defined 3 zones. Zone 1 referred to the emergence point of the LON from deep to or behind the SCM. Zone 2 was the cephalic ascent of the LON along or posterior to the SCM. Lastly, Zone 3 represented the crossing point of the LON at the nuchal line. They calculated the average perpendicular distances of Zone 1, Zone 2, and Zone 3 to the posterior midline as 6.3 ± 1.37 cm, 6.2 ± 1.10 cm, and 5.9 cm, respectively. Similarly, they found the average perpendicular distances of these points to the transverse line that passed through EOP to be 7.8 ± 1.84 cm, 5.5 ± 1.38 cm, and 3.8 cm, respectively. Tubbs et al. used 10 adult formalin-fixed cadaveric heads (20 sides)^12^. In their study, the LON was on meanly 6.8 cm lateral to the EOP. Shin et al. dissected a total of 35 formalin-fixed Korean cadavers (70 sides) and found that the LON crossed the lateral third of the line (an average distance of 33.9 mm) between the EOP and the lowermost point of the MP^17^. Khavanin et al. investigated the LON on seven fresh cadavers (14 sets of nerves) and reported that the perpendicular distance between the MP to the point where the LON arose from the posterior margin of the SCM was meanly 45.2 mm^2^. Amirlak et al.^18^ studied six cadaver necks and performed measurements similar to the reference lines (x and y axes) used by Lee et al.^15^. They calculated the mean perpendicular distances of the point where the LON arose from the posterior border of the SCM to the y axis and x axis were 7.5 ± 1.31 cm and 8.47 ± 1.11 cm, respectively. Additionally, they recorded the average distance from this point to the base of the lobule as 6.75 cm. In the present study, the perpendicular distance of the point where the LON emerged from the posterior border of the SCM to the vertical and transverse lines passing through the EOP was obtained as 63.69 ± 11.28 mm and 78.83 ± 17.21 mm, respectively. Our result of the perpendicular distance of the point to the vertical line passing through the EOP affirms those of the previous studies^3,12,15^. However, in our study, the perpendicular distance of the point to the transverse line passing through the EOP is not compatible with those of previous studies^3,12,15^. This discrepancy may be caused by the studies being conducted in different races and/or with different samples. Peled et al. noted that the point where the LON exits the posterior point of the SCM may be one of the most proximal points for the LON^5^. We believe that the morphometric values we obtained may serve as reference values for surgeons in surgical release/decompression surgery at this point. That is, we believe that the nerve may be easily identified/found along the posterior border of the SCM at a point approximately 6.5 cm lateral and 8 cm caudal to the EOP. Occipital nerve block can be used for the diagnosis and treatment of migraine, cervicogenic headache, and occipital neuralgia^2–4^. Being as close as possible to the target nerve during a block is important for the success of the block^19^. Dash et al. reported that this point was the ideal point to block the LON. In this context, we believe that our morphometric data may be used as a guide in the blocking process of LON^3^. There was a statistical significance between genders for the perpendicular distance of the LON to the vertical line passing from the EOP (*p* = 0.027). Saglam et al. obtained a similar result for the GON and reported that this may be due to the differentiation of the nerve for the genders during embryologic development^20^. We think that the statistical difference we obtained may be due to similar reasons. It can be argued that invasive procedures for the point where the LON emerged from behind the SCM may be significantly different between the sexes. That is, this point is significantly farther away to the midline in males than in females.

In the current study, the shortest distance between LON and GAN was meanly 16.62 ± 10.59 mm. GAN can be often used as a landmark to identify its nearby structures^2^. We think that the average value we obtained may help to find the emergence point of the LON behind the SCM, approximately 1.5 cm above GAN, using the emergence point of GAN behind the SCM as a reference. We also think that this value may be used as a reference value to avoid damaging the GAN during invasive procedures at the exit point of the LON. However, since this value varies between 0 and 43.40 mm, we believe that the LON and GAN may exit the nerve point of the neck (punctum nervosum) together, and in this case, more care should be taken when dissecting the LON to avoid damaging the GAN or vice versa for minimizing risk of injury and sensory deficits.

In our study, the linear distance of the LON from its emergence at the SCM to its branching point was meanly 31.24 ± 15.95 mm. We did not find any study in the literature evaluating such a parameter. Peled et al. reported that the first branching point occurred on average 3.8 cm caudal to the EOP and 5.9 cm lateral to the MP^5^. We think that the value we obtained may be helpful for surgeons in decompression surgery or nerve block applications planned to be performed for this point. In other words, this potential compression point of the LON may be easily reached at a point about 3 cm cranially the emergence point of the LON behind the SCM.

Collectively, our morphometric measurements may be important to avoid damage to the LON during a face-lift procedure^6^. Moreover, these measurements may be important in preserving LON in microvascular decompression surgery for hemifacial spasm. Thus, the risk of developing occipital sensory disturbance, a common complication, can be prevented^7^. Furthermore, there is a risk of injury to the LON during the retrosigmoid approach and surgeons should be aware of the course of this nerve^8^. Consequently, we believe that the measurements may reduce the risk of injury to the LON during the retrosigmoid approach.

*Limitation(s) of this study* The most important limitation is the relatively small sample size, but it can be said that our results are generally consistent with the literature. There are no data on the clinical presentation of our specimens. If they were available, it would be possible to determine whether a particular type having a particular clinical presentation.

## Conclusion

In this study, different types of the LON previously reported in the literature were observed together. Also, unlike the literature, the distance between the points where the LON and GAN emerged behind the SCM, and the distance between the point where the LON exited behind the SCM and the first branch of the LON, were determined. We obtained a statistical significance between genders for the perpendicular distance of the point where the LON emerged from the posterior border of the SCM to the vertical line passing from the EOP (*p* = 0.027). This means that this point is significantly farther from the midline in men than in women, and may indicate that invasive procedures for the point where the LON exits behind the SCM are significantly different between men and women.

As a conclusion, this study presents novel findings both morphological and morphometric features related to the LON. We believe that the morphological and morphometric data may aid clinicians in estimating the location of the nerve and/or its branches for block or decompression surgery as well as preservation of the LON during related procedures.

## Data Availability

The datasets generated during and/or analyzed during the current study are available from the corresponding author on reasonable request.

## Abbreviations

LON: Lesser occipital nerve
SCM: Sternocleidomastoid muscle
EOP: External occipital protuberance
GAN: Great auricular nerve
GON: Greater occipital nerve
IRB: Institutional Review Board
TM: Trapezius muscle
SC: Splenius capitis muscle
LS: Levator scapulae muscle
MP: Mastoid process
